# Deficient biological motion perception in schizophrenia: results from a motion noise paradigm

**DOI:** 10.3389/fpsyg.2013.00391

**Published:** 2013-07-04

**Authors:** Jejoong Kim, Daniel Norton, Ryan McBain, Dost Ongur, Yue Chen

**Affiliations:** ^1^Department of Psychology, Duksung Women's UniversitySeoul, South Korea; ^2^Department of Psychology, Boston UniversityBoston, MA, USA; ^3^Department of Global Health and Population, Harvard School of Public HealthBoston, MA, USA; ^4^Department of Psychiatry, Harvard Medical School, McLean HospitalBelmont, MA, USA

**Keywords:** biological motion perception, visual motion perception, bottom-up process, social cognition, schizophrenia

## Abstract

**Background**: Schizophrenia patients exhibit deficient processing of perceptual and cognitive information. However, it is not well-understood how basic perceptual deficits contribute to higher level cognitive problems in this mental disorder. Perception of biological motion, a motion-based cognitive recognition task, relies on both basic visual motion processing and social cognitive processing, thus providing a useful paradigm to evaluate the potentially hierarchical relationship between these two levels of information processing.

**Methods:** In this study, we designed a biological motion paradigm in which basic visual motion signals were manipulated systematically by incorporating different levels of motion noise. We measured the performances of schizophrenia patients (*n* = 21) and healthy controls (*n* = 22) in this biological motion perception task, as well as in coherent motion detection, theory of mind, and a widely used biological motion recognition task.

**Results:** Schizophrenia patients performed the biological motion perception task with significantly lower accuracy than healthy controls when perceptual signals were moderately degraded by noise. A more substantial degradation of perceptual signals, through using additional noise, impaired biological motion perception in both groups. Performance levels on biological motion recognition, coherent motion detection and theory of mind tasks were also reduced in patients.

**Conclusion:** The results from the motion-noise biological motion paradigm indicate that in the presence of visual motion noise, the processing of biological motion information in schizophrenia is deficient. Combined with the results of poor basic visual motion perception (coherent motion task) and biological motion recognition, the association between basic motion signals and biological motion perception suggests a need to incorporate the improvement of visual motion perception in social cognitive remediation.

## Introduction

Schizophrenia is characterized by deficits at multiple levels of information processing, including perception and cognition. Within the domain of visual perception, a large body of research indicates that patients with schizophrenia experience global dysfunction, particularly within the dorsal visual pathway (Butler and Javitt, 2005; Butler et al., 2008, 2012; Chen, 2011; Silverstein and Keane, 2011). For instance, they exhibit prolonged visual backward masking effects (Green et al., 1994), poor velocity discrimination (Chen et al., 1999; Kim et al., 2006; Clementz et al., 2007), and deficient global motion perception (Stuve et al., 1997; Chen et al., 2003; Green et al., 2009).

Difficulty in social interaction is another area of dysfunction in schizophrenia (Frith and Frith, 1999). While perception presumably impacts social functioning, whether and how visual perception deficits contribute to social dysfunction in schizophrenia has yet to be thoroughly investigated. To address this issue, it is important to identify and examine functional domains that intimately involve both visual and social cognitive processes. Perception of biological motion (BM) refers to visual recognition of other people's actions that are portrayed solely by motion signals [e.g., point-light animations, (Johansson, 1973)]. BM perception requires bottom-up integration of signals from basic visual motion perception along with top-down social cognition. For example, Neri and colleagues (1998) showed that when visual motion signals were degraded experimentally the perception of BM in healthy people collapsed (Neri et al., 1998), highlighting the importance of bottom-up processing in this task. Interestingly, other previous studies have also found that successful performance on BM perception tasks requires knowledge-based representations of biological organisms' typical actions. This type of evidence serves to reinforce the importance of top-down processing in BM perception (Dittrich, 1993; Thornton et al., 2002). The involvement of both perceptual and cognitive systems was also shown in a recent electrophysiological study which reported earlier and later peaks of EEG in response to BM from scalp sites corresponding to area MT, superior temporal sulcus (STS) and the parietal “mirror neuron system”(Krakowski et al., 2011). Similarly, brain imaging studies have revealed that the posterior superior temporal sulcus (pSTS) and some parietal areas are selectively activated during BM perception (Grossman et al., 2000; Grezes et al., 2001; Vaina et al., 2001b; Pavlova et al., 2004; Peuskens et al., 2005; Krakowski et al., 2011). Some frontal areas including inferior frontal sulcus (IFS) and premotor area are also activated during BM perception, which suggests an involvement of higher-order processing and attention (Saygin et al., 2004; Saygin, 2007).

In clinical domains, patients with neurodevelopment disorders such as autism showed abnormal performances in perception of BM (Blake et al., 2003; Annaz et al., 2010; Koldewyn et al., 2010). Not only in autism, impaired recognition and detection of BM have been found in schizophrenia (Kim et al., 2005, 2011; Singh et al., 2011). Schizophrenia patients also exhibited deficits associated with both basic visual motion processing such as detection of coherent motion (Chen et al., 2005) and with higher order social cognitive processing, such as theory of mind (ToM) tasks (Baron-Cohen, 1995; Frith and Frith, 1999). It is still unclear, however, whether impairment in recognition of BM in schizophrenia represents an extension of deficient basic motion perception (i.e., a bottom-up problem), impaired social cognitive processing (i.e., a top-down problem) or both.

In BM tasks, point-light animations are used to depict various types of actions—including walking, jumping, kicking, and running. To successfully distinguish biological motion from non-biological motion observers may rely on knowledge-based top-down cognitive processes as well as bottom-up perceptual processes. Thus, conventional biological motion tasks have not been designed to effectively determine whether perceptual factors or social cognitive factors contribute to impaired performance in schizophrenia during BM perception.

To examine the role of visual motion perception in determining BM perception ability, we designed a motion noise BM paradigm. In this paradigm human action (walking) that was presented as point-light dots was used to make up a part of the stimulus. Visual motion noise was then added as the other part of the stimulus. This paradigm is similar to many other paradigms which have been used previously for studying BM perception (Cutting et al., 1988; Neri et al., 1998; Thornton et al., 2002; Thompson et al., 2007; Garcia and Grossman, 2008). Motion noise in this paradigm served to modulate the perceptual signal strength, which is required for the performance of a BM task. By systematically changing the levels of noise, we were able to directly evaluate the effect of the perceptual signal strength on perception of BM. We call this motion noise task paradigm “perceptual discrimination of BM” (p-BM), due to its focus on the perceptual dimension of the task.

The relationship between basic visual motion perception and the perception of BM can also be evaluated by a comparison of respective performances on several associated tasks (e.g., detection of coherent motion vs. recognition of biological motion). In this study we measured performance on both a basic visual motion task—detection of coherent motion (Newsome and Wurtz, 1988)—and on another BM task—recognition of BM (Blake et al., 2003; Kim et al., 2005). We also measured performance in a widely used theory-of-mind task—the Eyes Test (Baron-Cohen et al., 2001)—in order to evaluate the relationship between perception of BM and higher-order social cognition. Comparison of performance among these tasks provided supplemental information on how basic perceptual and higher level social cognitive processes are related to the perception of BM.

## Materials and methods

### Subjects

Twenty-one patients with schizophrenia or schizoaffective disorder and twenty-two healthy controls participated in this study (Table 1). General inclusion criteria for both groups of participants were (1) between the ages of 18 and 55 years old, (2) no history of drug or alcohol abuse in the 6 months prior to participation, (3) no neurological problems such as seizure, stroke, or major head injury, and (4) Verbal IQ > 70.

**Table 1:** **Demographic information of participants**.

**Group**	**Patients**	**Controls**
Age (year)	39.1 (9.4)	36.7 (14.9)
Sex (M/F)	11/10	10/12
Education (year)	14.2 (2.6)	15.0 (2.4)

Patients were recruited when they responded to advertisements posted on the campus of McLean Hospital as well as in the Greater Boston area. Patient diagnosis was established using the Structured Clinical Interview for DSM-IV [SCID, (First et al., 2002)] which were administered by independent clinicians who were blind to the purposes of the study and by evaluating available medical records. Psychotic symptoms of patients were assessed with the Positive and Negative Symptom Scale (PANSS) (Kay et al., 1987). Their mean positive, negative, and general scores of the PANSS were 15.2 [standard deviation (*SD*) = 6.5], 13.4 (*SD* = 5.5) and 29.2 (*SD* = 8.6), respectively. Their average prescribed antipsychotic dose, calculated using the chlorpromazine equivalent (Woods, 2003), was 429 (*SD* = 361) mg.

Healthy controls (HC) were recruited when they responded to advertisements posted on the campus of McLean Hospital as well as in the Greater Boston area. They were screened for exclusion of psychiatric illness using the non-patient version of the SCID-IV (First et al., 2002).

The verbal component of the Wechsler Adult Intelligence Scale-Revised (WAIS-R) (Wechsler, 1981) was administered to all participants. The two participant groups were similar in age and education, but the patient group scored lower in verbal IQ when compared to the control group. All participants had normal or corrected-to-normal vision.

The study protocols were approved by the Institutional Review Board of McLean Hospital.

### Procedures

Participants performed all four tasks within the same research laboratory. All task procedures were implemented on a Macintosh G4 computer (Apple Inc. Cupertino, CA) which was placed in an otherwise dark room. Stimuli (see below for each task) were displayed on a ViewSonic CRT monitor GS 790 (ViewSonic Corp. Walnut, CA). A chinrest was used to stabilize the head of subjects and to maintain the viewing distance (57 cm). The entire procedure took approximately 40 min to complete. When needed, resting breaks were available between tasks.

#### Task 1: perceptual discrimination of biological motion (p-BM)

**Figure 1:**
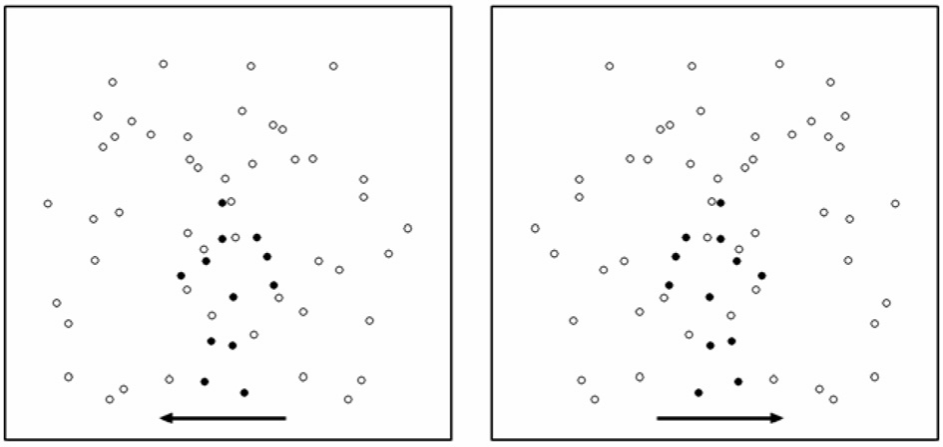
**An illustration of stimuli used in Task 1 (perceptual discrimination of BM)**. Here, for the purpose of illustration, signals dots are signified in a more salient color (dark). Noise dots are signified in a less salient color. The arrows indicate two possible walking directions (left and right). In an actual display, the two types of dot were in the same color and the arrows were not shown.

This task was specifically used to assess the perceptual capacity for discerning BM. The target was a point-light animation [12 dots on the head and major joints of the body; see details in (Blake et al., 2003)] of one type of BM—walking (leftward and rightward). The size of each dot was 5-arc min with the average speed within a sequence of 4°/s, and each sequence consisted of 20 frames. This target was embedded in a number of noise dots, and they together constituted a stimulus for perceptual discrimination of BM (Figure 1). The proportion of target dots in the BM stimulus is considered the perceptual signal; a large percentage of target dots provides a strong perceptual signal and therefore makes the task easier.

Noise dots consisted of duplicated dots from the original biological walkers (Cutting et al., 1988; Saygin et al., 2010), but with the following manipulations applied: half of the motion paths of the noise dots were generated from a walker moving rightward, whereas the other half were generated from a walker moving leftward. The makeup of these noise patterns was different from many previous paradigms in which the noise was derived from just one particular type or direction of action such as the movement of a leftward or a rightward walker alone. This “half and half” methodological modification allowed our paradigm to provide a more balanced noise profile in terms of randomness of motion direction and also allowed us to adjust signal strength at basic visual motion processing levels. The starting points of the noise dots were repositioned to random spatial locations.

This perceptual discrimination paradigm used only one type of BM (walking) and a simple task of direction discrimination to minimize the requirement for knowledge-based top-down information about a variety of prototypes of the BM. That is, participants did not have to reallocate extra attentional resources to determine whether one of many BMs or a scrambled motion would appear. Rather, since a walker was always present, participants could simply focus on discriminating between two walking directions by interpreting the kinematical information in the spatiotemporal pattern of the stimulus. As a result, performance on this BM task relies primarily on the bottom-up process based motion perception.

The stimulus was generated within MatLab's (Mathworks Inc., Natick, MA) Psychophysics Toolbox (Brainard, 1997; Pelli, 1997) programming environment. The entire array of dots, including noise dots, had a size of 7 × 7° in visual angle, and was displayed for 1 s in each trial. The size of the target (walker) was approximately 4° (height)× 3°(width). Participants indicated the direction of walking (leftward or rightward) by pressing one of the two pre-assigned buttons, and were instructed to guess when necessary. The total number of noise dots varied between 12, 24, 48, 96, or 192. These five noise levels corresponded to five signal-to-noise ratios for the stimulus—100.0%, 50.0%, 25.0%, 12.5%, and 6.3%. The different signal-to-noise ratios were presented across trials according to the method of constant stimuli. Each combination of noise condition and walking direction was repeated 10 times. The total number of trials was 100. Compared to other methods (e.g., QUEST, a staircase threshold estimation procedure), the method of constant stimuli is more thorough but less efficient, but allowed us to show the stimulus conditions (or noise level) under which the performance of patients and the performance of controls do and do not differ. The data generated from this method provide an illustration of the pattern of performance change with stimulus condition.

One performance measure we utilized was the accuracy with which participants identified the moving direction of the walker presented at each of the five signal-to-noise ratios. Another performance measure was the participant's perceptual threshold, which was defined as the maximum noise level at which participant's performance reached 80% accuracy. Unlike many perceptual threshold metrics, for the purposes of this study a higher threshold value corresponds with a better performance.

#### Task 2: detection of coherent motion (CM)

This task has been widely used to assess the capacity of perceptual processing of visual motion information (Newsome and Wurtz, 1988), including in patients with neurological disorders (e.g., Vaina et al., 2001a) and schizophrenia (Stuve et al., 1997; Li, 2002). The stimulus consisted of signal dots moving coherently toward one direction (left or right), and noise dots moving in random directions. Those two portions of dots were intermixed and randomly distributed in space through a circular window. The proportion of signal dots (coherence level) in the random dot pattern (RDP) is considered the motion signal strength or task difficulty; a larger percentage of the signal dots corresponds with a stronger motion signal and therefore makes the task easier.

The stimulus was generated within the C programming environment. The stimulus had a size of 7° in diameter. In each trial, an RDP was presented for 400 ms. Participants indicated the direction of coherent motion (left or right) by pressing one of two designated keys, guessing when necessary. Six levels of motion coherence (0, 5, 10, 20, 40, and 100%) were presented in a random order across trials according to the method of constant stimuli. Performance was measured in two ways: (1) the participant's response accuracy at each of the six motion coherence level, (2) his/her perceptual threshold, which was defined as the minimum coherence level at which participant's performance level reached 80% accuracy. A lower threshold value corresponds with a better performance.

#### Task 3: recognition of BM (r-BM)

This task was used to assess a participant's ability to discriminate BM from non-biological, scrambled motion. The BM recognition task has been used in various clinical populations including patients with autism (Blake et al., 2003), schizophrenia (Kim et al., 2005) and obsessive-compulsive disorder (Kim et al., 2008), as well as in infants (Hirai and Hiraki, 2005). Stimuli consisted of point-light animations depicting various prototypes of human actions (e.g., walking, kicking, jumping, throwing, and so on), and their spatially scrambled versions. The scrambled versions were generated by randomizing the initial positions of each dot in their corresponding point-light BMs (Grossman et al., 2000; Blake et al., 2003).

The stimulus was generated within the MatLab/Psychophysics Toolbox programming environment. The stimulus had a size of approximately up to 6°(height) × 4°(width) in visual viewing angle and was displayed for 1 s in each trial. The biological and scrambled motions were presented in a random order across trials. The task was to indicate whether a given stimulus in each trial was a biological or scrambled motion by pressing one of two pre-assigned keys. There were 25 prototypes of BM, each of which had two facing directions, comprising 50 different BMs in total. The total number of trials was 100, including 50 presentations of BM and 50 presentations of scrambled motion.

Participants could not predict which type of action would be presented for each trial. Performance on this task thus relied not only on perceptual processing of motion signals, but also on knowledge-based cognitive representations of biological action prototypes.

Performance was measured by discrimination sensitivity (*d*'), which is defined as the difference of standardized “hits” (BM responses to BM stimuli) and standardized “false alarms” (BM responses to scrambled motion stimuli). A higher value of discrimination sensitivity corresponds to a better performance.

#### Task 4: eyes test

This task was used to assess the ability of participants to recognize emotional expressions of other people based upon an image of the eye region of their face (Baron-Cohen et al., 2001). This ability is closely associated with theory of mind (ToM) of social functioning. The original Eyes Test (the revised version of the “Reading the Mind in the Eyes” task) was in paper version. For this study, the Eyes Test was converted to an electronic version; the images were displayed on a computer screen within the MatLab/Psychophysics Toolbox programming environment. There were 36 images, and each image remained on the screen until a response from the participant was registered. The task was to view each image and then choose a word (out of four options) that best described what the person in the given image was feeling or thinking.

Performance was measured by the proportion of responses for which the word that correctly describes an emotional expression was chosen.

## Results

### Perceptual discrimination of biological motion (p-BM)

**Figure 2:**
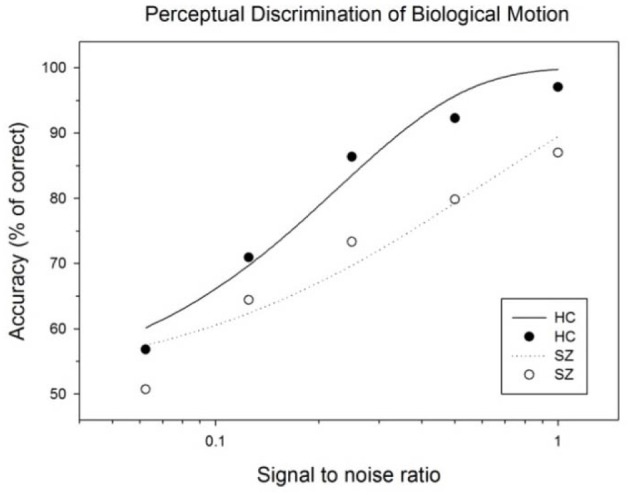
**Subject performances as a function of signal to noise ratio of BM stimulus**. The curves represent the fits of data by a psychometric function (Weibull equation) for each subject group.

A Two-Way ANOVA (group × signal strength) on performance accuracy showed a significant main effect for signal strength [*F*_(4, 160)_ = 103.10, *p* < 0.001] and for group (1, 40) = 7.76, *p* = 0.008). The interaction effect between group signal strength was not significant [*F*_(4, 160)_ = 1.90, *p* = 0.11]. This study had limited statistical power due to the moderate sample size, therefore we have chosen to compare the group differences for each signal strength (noise) level. *Post-hoc* tests showed that the performance accuracies of schizophrenia patients were significantly lower than those of healthy controls when perceptual signals were moderately degraded (*p* = 0.006, Cohen's *d* = 0.88 for 100.0%; *p* = 0.005, Cohen's *d* = 0.91 for 50.0%; and *p* = 0.012, Cohen's *d* = 0.81 for 25.0%)^1^. When perceptual signals were more substantially degraded, the performances of the two groups did not differ significantly (*p* = 0.13, Cohen's *d* = 0.48 for 12.5%; and *p* = 0.19 Cohen's *d* = 0.44 for 6.3%) (Figure 2) and (Table 2).

**Table 2:** **Performance accuracy in p-BM task: group mean (*SD*)**.

	**100% (12 noise dots)**	**50% (24 noise dots)**	**25% (48 noise dots)**	**12.5% (96 noise dots)**	**6.25% (192 noise dots)**
Control	97.05 (7.66)	92.27 (13.15)	86.36 (13.73)	70.91 (14.19)	56.81 (11.7)
Patient	87.0 (14.18)	79.83 (14.19)	73.32 (18.31)	64.41 (12.86)	50.72 (17.82)

**Figure 3:**
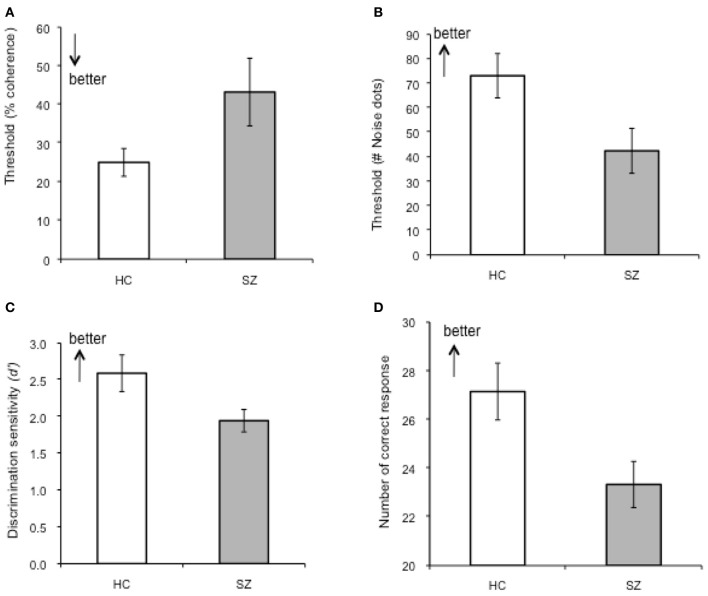
**Summary of the performances on basic visual motion perception and BM perception tasks. (A)** Perceptual threshold for coherent motion detection (CM), **(B)** Perceptual discrimination threshold of BM (p-BM), **(C)** Discrimination sensitivity for recognition of BM (r-BM). **(D)** Accuracy for the Eyes Test. Error-bars indicate one standard error (SE).

Perceptual discrimination thresholds, defined as a maximum noise level at which participant's performance maintained at 80% accuracy, were 73.14% (*SD* = 42.36%) for controls and 42.51% (*SD* = 40.96%) for patients, which yielded a significant group difference (*p* = 0.02, Cohen's *d* = 0.74) (Figure 3B). This result indicates that schizophrenia patients tolerated a smaller number of noise dots than healthy controls in order to adequately perform the task.

When verbal IQ score, the only behavioral variable differing between patients and controls, was used as covariate, an ANOVA on performance accuracy yielded similar results to the original analysis [*F*_(4, 156)_ = 2.78, *p* = 0.03 for signal strength, *F*_(1, 39)_ = 5.31, *p* = 0.03 for group, and *F*_(4, 156)_ = 1.44, *p* = 0.22 for interaction].

### Task 2: detection of coherent motion (CM)

A Two-Way ANOVA (group × signal strength) on performance accuracy showed a significant main effect for signal strength [*F*_(5, 165)_ = 25.92, *p* < 0.001] and for group (1, 33) = 4.42, *p* = 0.04). The interaction effect between group signal strength was not significant [*F*_(4, 165)_ = 1.33, *p* = 0.25]. Performance accuracies of schizophrenia patients were lower than those of healthy controls, yet *post-hoc* tests showed that the group differences did not reach a conventional statistical criterion level at each motion coherence condition (except for the 100% condition).

Perceptual thresholds for detecting coherent motion (the minimum coherence level guaranteeing 80% accuracy) were higher (lower performance level) in patients than in controls [controls: 25.05 (*SD* = 16.05), patients: 43.25(*SD* = 36.06)]; the group difference was marginally significant (*p* = 0.049, Cohen's *d* = 0.65). That is, compared to controls, patients required a higher level of motion coherence or stronger motion signals in order to reach the designated performance accuracy level (80%) (Figure 3A) and (Table 3).

**Table 3:** **Accuracy for coherent motion detection at each coherence condition: group mean (*SD*)**.

	**0%**	**5%**	**10%**	**20%**	**40%**	**100%**
Control	49.68 (14.68)	55.52 (12.37)	71.04 (11.02)	76.97 (14.63)	89.79 (11.74)	98.43 (3.43)
Patient	53.35 (13.08)	50.39 (10.57)	64.06 (16.84)	70.93 (24.31)	80.85 (26.75)	92.57 (10.51)
*p*-value	0.44	0.19	0.14	0.36	0.19	0.025^*^
Effect size (Cohen's *d*)	0.34	0.44	0.49	0.30	0.43	0.75

* *p* < 0.05.

When the verbal IQ score was used as a covariate, an ANOVA yielded non-significant effects for signal strength [*F*_(5, 160)_ = 2.18, *p* = 0.059], and group [*F*_(1, 32)_ = 2.24, *p* = 0.14], both of which differed from effects for the original analysis and in terms of the interaction effect [*F*_(5, 160)_ = 1.08, *p* = 0.37].

### Task 3: recognition of biological motion (r-BM)

The discrimination sensitivity of patients was significantly lower than that of controls (patients: *d*' = 1.94 (*SD* = 0.69), controls: *d*' = 2.59 (*SD* = 1.16), *p* = 0.03, Cohen's *d* = 0.68) (Figure 3C). Separate analyses of the hit rates and false alarm rates, the two components of discrimination sensitivity, revealed that the poor performance of patients was mainly due to their high false alarm rates [hits: *t*_(41)_ = 0.52, *p* = 0.61; false alarm: *t*_(41)_ = −2.68, *p* = 0.01]. The resulting false alarm rate indicated that patients were more likely to attribute non-biological, scrambled motion as BM, compared to controls. This result replicated one of our previous findings (Kim et al., 2011).

When the verbal IQ score was used as a covariate, an ANOVA yielded non-significant group effects in *d*' [*F*_(1, 40)_ = 3.38, *p* = 0.072] and hit rate [*F*_(1, 40)_ = 0.29, *p* = 0.59], and a significant group effect in false alarm [*F*_(1, 40)_ = 6.16, *p* = 0.017].

### Task 4: the eyes test

The performance accuracy of the patient group was significantly lower than that of the control group [*t*_(39)_ = 2.55, *p* = 0.015, Cohen's *d* = 0.79] (Figure 3D). For the total 36 images, the group mean (*SD*) of correct responses of the controls and of the patients were 27.15 (5.22) and 23.33 (4.34), respectively. This result confirms deficient social functioning in the patient group.

When the verbal IQ score was used as a covariate, an ANOVA yielded a non-significant effect for group [*F*_(1, 38)_ = 3.38, *p* = 0.074].

### Relationship of the performances in these tasks

We used Pearson's correlation to evaluate the relationships among performances of the four tasks. The analysis results are summarized in Table 4.

**Table 4:** **Correlations between the performances on the visual and cognitive tasks used in this study**.

		**CM**	**p-BM**	**r-BM**	**r-BM (hit)**	**r-BM (false alarm)**	**Eyes test**
Controls	CM						
	p-BM	−0.28					
	r-BM	−0.06	0.395				
	r-BM(hit)	−0.10	0.534^*^	0.548^**^			
	r-BM(false alarm)	−0.47^*^	0.22	0.109	0.377		
	Eyes test	−0.507^*^	−0.09	0.003	0.176	0.263	
Patients	CM						
	p-BM	−0.307					
	r-BM	−0.623^**^	0.514^*^				
	r-BM(hit)	−0.31	0.44	0.607^**^			
	r-BM(false alarm)	0.36	−0.005	−0.462^*^	0.322		
	Eyes test	−0.66^**^	0.505^*^	0.43	0.39	−0.088	

CM, Detection of coherent motion; p-BM, perceptual discrimination of biological motion; r-BM, recognition of biological motion.

* *p* < 0.05,

** *p* < 0.01.

In the patient group, CM was significantly correlated with r-BM (*r* = −0.62) and the Eyes Test (*r* = −0.66). There was a moderate, yet non-significant correlation between CM and p-BM (*r* = −0.31).

### Relationship between the task performances and other clinical variables

In the patient group, neither positive nor negative PANSS scores were correlated with performances on perceptual tasks. Likewise, CPZ was not correlated with performance on any tasks.

Across all participants verbal IQ was significantly correlated with Eyes Test scores (*r* = 0.41, *p* = 0.008). Since the patients had lower scores in both the Eyes Test and verbal IQ compared with healthy controls, the significant correlation across all participants may be due to diagonal alignment of two clusters rather than a truly linear relationship. In the patient group alone, the correlation between verbal IQ and the Eyes Test was 0.42 (*p* = 0.062).

### Additional testing of the p-BM in a subgroup of participants

As a follow-up, we assessed performance on the main task (p-BM) under a no-noise condition in a subgroup of participants (*n* = 8 for patients and *n* = 7 for controls). This no-noise condition, while not included during the initial testing, provides a baseline for comparisons with those conditions containing various levels of noise. For this condition, the performance accuracy was 100% (*SD* = 0%) for healthy controls and 99.38% (*SD* = 1.77%) for the patients. The group performances did not differ significantly (*p* = 0.37).

## Discussion

The results of this study show that patients performed significantly worse at BM perception in the presence of visual motion noise. Compared to controls, perceptual discrimination of BM in the patients was impaired when perceptual signals were moderately degraded by motion noise. When perceptual signals were more substantially degraded, perceptual discrimination of BM was similarly impaired in both groups. This study also replicated previous results of deficient basic visual motion perception (Li, 2002; Chen et al., 2005; Slaghuis et al., 2007), deficient recognition of BM (Kim et al., 2005, 2011) and poor theory of mind (Frith and Corcoran, 1996; Abu-Akel, 1999) in this patient population. In the following sections, we consider the mechanisms that may contribute to deficient BM perception in schizophrenia.

### Bottom up processes for biological motion perception

Performance in a BM task requires extraction and recognition of a prototype of human action from dozens of possibilities. Such a requirement involves information processing from both bottom-up and top-down systems. For example, a knowledge base about possible BMs must exist and be accessed, which is a top-down component important for recognition of these human actions. Thus, both bottom-up and top-down processes may be implicated in patients' impairments on the r-BM task.

Compared to the r-BM task, perceptual discrimination of BM (p-BM) restricts itself to a single prototype of human action (walking) and evaluates a simple visual feature of the action (walking direction). Such a design minimizes the influence of top-down cognitive processes like the retrieval of information from one's knowledge base about various other types of BM. Importantly, the manipulation of the independent variable—motion noise—in this design is stimulus-based and does not involve top-down processes. For this reason changes in subjects' performance as a result of changes in this variable cannot be attributed to top-down abilities. Patients' poor performance on this task primarily implicates the bottom-up processes that support BM perception.

This vulnerability of BM perception to motion noise is not necessarily associated with a specific disease process. Children (6 years old and younger) under normal perceptual and cognitive development showed immature performances when motion noise was present (Freire et al., 2006). Adverse effects of such noise masking also appeared when adolescents with autism performed on perception of BM (Koldewyn et al., 2010) [but also see (Saygin et al., 2010)]. Along with the degraded performance on the CM task in the present study and in previous studies (Stuve et al., 1997; Chen et al., 2003), patients' poor performance on the p-BM task may reflect a problem of bottom-up processing in this disorder.

### Effects of perceptual modulation on biological motion perception

BM perception of patients and controls was substantially and comparably impaired in the presence of a high level of motion noise (96–192 dots, Figure 2). For a moderate level of motion noise (12–48 dots), however, patients' performance was significantly more degraded than that of controls. For the no-noise condition, the data from a subgroup of participants shows perfect or nearly perfect performance in both controls and patients. The results of degraded BM perception in the presence of visual motion noise may be interpreted in two ways. First, patients' lower performance level for the moderate noise conditions may be due to their deficient processing of BM information which is evident across all stimulus conditions and may be irrelevant to the presence of noise. This interpretation would be consistent with the existence of a generalized deficit in BM perception for patients even when no noise is present. Second, impairments in patients' performance at moderate noise levels could be particular to the presence of noise. This interpretation would suggest that patients and controls would have shown similar performance when no noise is present. The additional testing of patients (*n* = 8) and controls (*n* = 7) in this no-noise condition did in fact show this type of similar performance between the two groups, suggesting that the latter interpretation is the more likely scenario. The result from the r-BM task casts further light on the subject. In the r-BM task, one aspect of performance—hit rate—is analogous to the performance accuracy under the no noise condition of p-BM task. Both of these performance indexes measured the detection of a BM while no irrelevant stimuli (such as noise) were present. The result of a similar hit rate in patients and controls in the r-BM task (Task 3 in the Result section) is consistent with a data collected in a small group of additional patients and controls which showed similar performance while no noise was present. The sensitivity of the patient group to visual motion noise in these tasks highlights the role of visual motion signals in the processing of BM.

### Relationships with basic visual perception

Given the putative relationship between basic visual processing and high level cognitive processing, one may consider that patients' deficit in BM perception is closely related to the deficit in basic visual motion perception. Both a non-significant correlation (p-BM vs. CM: *r* = −0.31) and a significant correlation (r-BM vs. CM: *r* = −0.62) were found in this study. This latter result is similar to that from a previous study in which a significant correlation between patients' performances on a CM task and on a BM recognition task was found (Brittain et al., 2011). The mixed correlation result is generally consistent with the notion that basic motion perception and BM perception may engage different cortical mechanisms (Vaina et al., 1990; Poom and Olsson, 2002).

An alternative interpretation would consider the role of form information. Random dot patterns, used in CM, do not contain any explicit or implicit form (except for the superimposed circular aperture which simply served as boundary). Although point-light animations, used in p-BM, do not have explicit form, spatiotemporal kinematics does draw form information of body shape. A recent study suggested that there are two critical features for precise perception of point-light walkers: upper body structure (form) and limb movements crossing each other (motion) (Thurman et al., 2010). Another recent study reported that observers were able to perceive global motion but not able to discriminate walking direction of BM when structural information was eliminated and motion information was intact (Lu, 2010). In this context, performance in CM task would only require integration of local motion signals into global motion while performance in p-BM task would require both a local motion integration and processing of implied form information as suggested by Giese and Poggio's model (Giese and Poggio, 2003). Therefore, schizophrenia patients' poor performance on p-BM task may implicate compromised processing of form information in addition to deficient motion processing (Takahashi et al., 2010). If this were the case, patients' performances on the two tasks should be partially correlated. Note that the interpretation of additional form processing for BM should be applicable to the relationship between the performances in CM and r-BM. The stronger correlation between patients' performances in the latter two tasks (*r* = −0.62) discounts this form processing interpretation.

### Altered cognitive processes

BM perception requires attention (Cavanagh et al., 2001; Wang et al., 2011). A general attention problem may affect patients' performance on BM tasks. However, such an attention problem cannot be a primary factor here, as patient performance under different task conditions would be similarly degraded if driven by a gross attention deficit. This was not the case in this study, where patients were differentially impaired at moderate noise levels. Patients also seemed differentially impaired among different tasks. For example, after IQ was used as covariate, the large group difference in the performance of p-BM task remained whereas the moderate group difference in the performance of CM task as well as Eyes Test became non-significant. This non-significance also does not favor a general attention deficit interpretation.

One may wonder about the role of general intelligence in determining performance in these groups, especially in light of results from autism research which has shown that IQ can predict perceptual ability on BM tasks (Rutherford and Troje, 2012) and account for perceptual deficits on CM tasks (Koldewyn et al., 2010). In the present study, the verbal IQ of schizophrenia patients was not significantly correlated with BM but explained about 20% of the variability in the BM task, suggesting a relationship between the two tasks whose nature (e.g., spurious vs. causal, direction of causality if present) is undetermined. Degraded IQ is generally considered *an inherent part of schizophrenia*, but nevertheless our result showed that when this IQ variable was adjusted, patients' deficient performance remained on the BM tasks but not on the CM or the Eyes Test. This result suggests that a selective deficit in processing biological motion information exists in this mental disorder.

Despite remarkable phenomenological differences between autism and schizophrenia, an overlap of aberrant biological processes is suggested by recent research (e.g., McCarthy et al., 2009; Crespi et al., 2010). This overlap may be reflected in the presence of similar impairments beginning at more basic behavioral levels such as basic visual motion perception in both of the disorders (Stuve et al., 1997; Spencer et al., 2000; Chen et al., 2003; Koldewyn et al., 2010) [but also see (White et al., 2006; Chen et al., 2012)]. However, whether or not the problems of BM perception in autism and schizophrenia are of the same nature is unclear. In autism, it has been suggested that high level cortical processes are to blame for this impairment (Saygin et al., 2010; Koldewyn et al., 2011). In schizophrenia, the results of this study have shown that deficits in BM perception were related to basic visual motion perception. Just how basic visual processing problems are associated with impairments in high level cognitive processes (such as BM perception or IQ) remains a topic for further explorations, as this effect seems to differ between autism and schizophrenia.

### Implications on general visual and cognitive processes

Recent studies have found an increased effect of visual modulation on other perceptual and cognitive processes in schizophrenia. One of our studies showed that patients' cognitive control of visually bi-stable images was more significantly influenced by the contrast level of visual stimulus than in controls (McBain et al., 2011b). Another study showed that patients' emotion perception (fear and happiness) was more substantially changed by a manipulation of spatial frequency of facial images (McBain et al., 2011a). It has also been shown that surrounding visual context influenced the action of finger-reaching toward a central target to a greater extent in patients than in controls (Chen et al., 2011). Along the same lines, this study found greater degradation of BM perception by visual motion noise in patients. The implications of increased interaction between basic visual motion perception and BM perception in schizophrenia are not immediately clear. But given the presence of deficient basic visual motion signal in this mental disorder, a stronger connection from basic motion processes to BM processes would be needed in order to utilize such weakened visual inputs during BM perception. Increased connectivity between the two levels of visual and cognitive processing may serve as one compensatory strategy.

### Cortical processing for biological motion perception

The processing of BM information is primarily mediated in the superior temporal sulcus [for review, see Blake and Shiffrar (2007), Pavlova (2012)]. It has been shown that patients' cortical responses in this area were not selective to biological or scrambled motion (Kim et al., 2011). Given that schizophrenia is a brain disorder involving many cortical systems, it is important to ask if other cortical systems could potentially contribute to the processing of BM information. Currently, no neuroimaging data are available to directly address this question. The behavioral data of this study support the notion that BM processing in schizophrenia is more sensitive to signal modulation in the basic visual domain. A key test for this notion is to what extent the visual cortical areas such as MT and STS are functionally connected in patients. If the processing in the basic motion system contributes to a greater extent in higher level processing in the BM system as suggested by the behavioral data of this study, then increased functional connectivity between the two anatomically separate systems should exist in this mental disorder. A recent study on functional connections of cortical systems during resting states found that while local functional connectivity was reduced, global distances of functionally connected brain areas, or “connection distance,” were increased in schizophrenia (Alexander-Bloch et al., 2013). The greater global functional connectivity suggested by this result is generally consistent with the suggestion that there may be heightened interaction among separate cortical systems (e.g., the visual cortex vs. the STS) in this mental disorder. A direct measurement of functional connectivity between the areas of interests would more definitely describe the relationships between basic visual processing and BM processing.

### Visual processing and social functioning

How basic visual processing deficits contribute to poor social functioning in schizophrenia is an important topic in schizophrenia research as this relationship could help inform the strategies of cognitive interventions. Based on a series of correlations among visual motion perception, BM perception and social cognition, Brittain et al. suggested that the processing of BM may act as an intermediate between visual perception and social behaviors (Brittain et al., 2011). Our study showed that patients' visual motion perception and bottom-up process driven BM perception (p-BM) were correlated with BM recognition (r-BM) which is supported by both bottom-up and top-down processes. Visual motion perception and the p-BM were also correlated with theory of mind, another aspect of social cognition. These results highlight the role of basic visual motion processing in socially meaningful tasks. Such a functional relationship suggests that the problems of processing visual and social cognitive information are likely associated in schizophrenia.

It is intuitive that the performances in BM tasks and in other social cognitive tasks like the Eyes Test are correlated. It is also intuitive that performances in the coherent motion and in the Eyes Test are correlated to a lesser extent, as the latter does not seem to be motion-related. The results of this study suggested otherwise: the correlation between perception of BM and the Eyes Test is lower than that between coherent motion detection and the Eyes Test. One way to understand such a result is to consider that the social brain receives common perceptual inputs and engages in two separate social cognitive processes, one dealing with dynamic signals (BM perception) and the other dealing with static signals (face processing, including signals involved in the Eyes Test). The common perceptual inputs, including those from coherent motion, feed into both social cognitive processes. Upon such a scenario, one would expect a robust correlation between the performances on coherent motion and the Eyes Test. Assuming that there are separate operations between dynamic and static social cognitive processes, one would also expect a weaker correlation between performances on BM and the Eyes Test.

Like a previous study (Kelemen et al., 2005), we found that patients' performances in CM and the Eyes Test were significantly correlated. This suggests that deficient visual motion processing in this mental disorder not only impacts motion-based but also non motion-based social cognition. The correlation between the performances in CM and the Eyes Test (*r* = −0.66) was as robust as the correlation between the performances in CM and r-BM (*r* = −0.62), which suggests that there are similar functional connections from the visual motion system to the social cognitive systems mediating BM perception and theory of mind.

Other visual perception deficits in schizophrenia may relate to social functioning problems differently. For example, Sergi and Green (2003) showed weak correlations between visual masking deficit and social functioning problems in patients. One may hypothesize that higher level visual processing like motion perception should be more strongly linked to social behavioral outcomes because this processing taps into social cognition. This hypothesis, while plausible, remains to be thoroughly tested by more systematic investigation.

### Limitations

One limitation of this study is that the data from the no-noise condition for the p-BM task were available only from a subgroup of participants. A more complete set of data would serve as a firm basis for comparison with those obtained under the noise conditions, and would more definitively indicate whether patients' degraded task performance under the noise conditions is due to the visual motion noise factor.

Another limitation is the use of medicated patients. It is difficult to exclude a medication effect on patients' visual and cognitive performances (e.g., Allen et al., 1997; Purdon et al., 2000). Yet, in this patient group, the CPZ dose equivalent was not correlated with the performance in any of the tasks used in the study. This suggests a minimal, if any, role of antipsychotic medications in the visual and cognitive tasks.

Still another limitation was the presence of a group difference in verbal IQ. It seems that when verbal IQ was taken into account in analyses the group differences remained for the BM task, but not for the CM task or the Eyes Test. Such a pattern of results points to a complex relationship between verbal IQ and perceptual/social cognitive measures. Given that the performance of high level cognitive tasks (e.g., the Eyes Test) relies upon verbal information, patients' low verbal IQ performance could potentially be a confounding factor when inspecting group differences in performances on social cognitive tasks. These limitations call for the results of this study to be verified with independent methods in future studies.

To summarize, this study found that in the presence of visual motion noise, BM perception was more substantially degraded in schizophrenia. Combined with patients' deficient performances in and relationships among basic motion perception, BM perception and theory of mind, the results of this study suggest that basic motion processing in schizophrenia plays an increased role in BM perception. The functional relationships between different levels of information processing highlight the importance of including methods for improvement of reduced visual motion perception capacity for social cognitive remediation in this mental disorder.

### Conflict of interest statement

The authors declare that the research was conducted in the absence of any commercial or financial relationships that could be construed as a potential conflict of interest.

## Footnotes

^1^With Bonferroni correction for multiple comparisons under the five stimulus conditions (*p* < 0.01), the group differences remained significant for the conditions of 100.0% (*p* = 0.006) and 50.0% (*p* = 0.005), and became statically non-significant for the 25.0% condition (*p* = 0.012).
